# Detection of SARS Coronavirus in Patients with Suspected SARS

**DOI:** 10.3201/eid1002.030610

**Published:** 2004-02

**Authors:** Kwok H. Chan, Leo L.L.M. Poon, V.C.C. Cheng, Yi Guan, I.F.N. Hung, James Kong, Loretta Y.C. Yam, Wing H. Seto, Kwok Y. Yuen, Joseph S. Malik Peiris

**Affiliations:** * Queen Mary Hospital, Hong Kong Special Administrative Region, People’s Republic of China (SAR); † University of Hong Kong, Hong Kong SAR; ‡ Hospital Authority Head Office, Hong Kong SAR; § Pamela Youde Nethersole Eastern Hospital, Hong Kong SAR

**Keywords:** SARS, severe acute respiratory syndrome, SARS coronavirus, virus, epidemiology, transmission, diagnosis, pathogenesis, research

## Abstract

Cases of severe acute respiratory syndrome (SARS) were investigated for SARS coronavirus (SARS-CoV) through RNA tests, serologic response, and viral culture. Of 537 specimens from patients in whom SARS was clinically diagnosed, 332 (60%) had SARS-CoV RNA in one or more clinical specimens, compared with 1 (0.3%) of 332 samples from controls. Of 417 patients with clinical SARS from whom paired serum samples were available, 92% had an antibody response. Rates of viral RNA positivity increased progressively and peaked at day 11 after onset of illness. Although viral RNA remained detectable in respiratory secretions and stool and urine specimens for >30 days in some patients, virus could not be cultured after week 3 of illness. Nasopharyngeal aspirates, throat swabs, or sputum samples were the most useful clinical specimens in the first 5 days of illness, but later in the illness viral RNA could be detected more readily in stool specimens.

In early 2003, severe acute respiratory syndrome (SARS) was recognized as a newly emerging pneumonic disease (*1*–*3*). A proportion of patients have watery diarrhea, usually at a later stage of the illness, suggesting that the infection may not be confined to the respiratory tract (*4*). A novel coronavirus, designated as SARS coronavirus (SARS-CoV), was implicated as the causative agent (*5*–*7*), and the respiratory disease has been reproduced in a nonprimate animal model (*8*). Hong Kong was one of the regions most affected, with >1,700 patients. Specific laboratory tests to detect viral RNA and antibody responses (*5*) were used to establish a cause in patients suspected to have SARS. Although virologic results for small cohorts of patients have been reported (*4*,*5*,*9*), analysis of results of these first-generation tests in routine clinical practice has not been published previously. We report the correlation of results of reverse transcriptase polymerase chain reaction (RT-PCR) and immunofluorescent serologic testing for SARS-CoV in 1,048 cases investigated for SARS in the first 5 weeks after the first-generation diagnostic tests became available in Hong Kong.

## Methods

### Patients

In the weeks after the first-generation viral diagnostic tests became available in Hong Kong, SARS-CoV diagnosis was carried out in three laboratories, one of which was the Department of Microbiology of Queen Mary Hospital (QMH). Results from specimens investigated at QMH laboratory from April 1 through May 3, 2003, and subsequent follow-up specimens are included in this analysis. Clinical specimens used for viral RNA detection included nasopharyngeal aspirates, throat and nose swabs, saliva, sputum, endotracheal aspirates, feces, and urine. Nasopharyngeal aspirates were collected into a mucus trap, and residual secretions in the catheter were sucked into the trap by aspirating 2 mL of virus transport medium. Swabs were collected into 2 mL of virus transport medium containing vancomycin (final concentration 100 μg/mL), amikacin (30 μg/mL), and nystatin (40 U/mL). Urine and feces were collected into specimen containers and submitted directly to the laboratory without the addition of transport medium.

The case definition has been previously described (*5*,*10*). Patients were categorized on a clinical basis as “clinical SARS,” “suspected SARS,” and “not SARS” by the attending clinicians, depending on the response to antimicrobial therapy for bacterial pathogens (e.g., tazocin 2.25–4.5 g intravenously 6–8 h/d, or azithromycin 500 mg/day for 7–10/d), the clinical and radiologic evolution of the illness, history of contact with other patients with SARS, and an alternative diagnosis that fully explained the clinical findings.

Fecal, throat swab, and serum specimens from controls were obtained for comparison. Fecal specimens from patients with diarrhea were anonymously tested for SARS-CoV RNA. Throat swab specimens were collected after informed consent from patients attending primary care facilities for nonrespiratory diseases and tested for SARS-CoV RNA. Blood donor sera left over from screening for bloodborne viruses were tested anonymously for antibodies to SARS-CoV.

### Viral RNA Detection

RNA extraction was performed by using QIAamp Viral RNA kit reagents (Qiagen, Hilden, Germany) according to the manufacturer’s instructions. The RT-PCR primers and conditions have been described (*5*,*11*). Since these primers gave occasional false-positive reactions with stool specimens, all PCR-positive stool specimens were retested by the LightCycler PCR (Roche Diagnostics GmbH, Mannheim, Germany) for confirmation using the same two sets of primers, with the melting curve analysis being used to provide additional confirmation of reaction specificity (*9*). A plasmid vector pCRII-TOPO (Invitrogen, San Diego, CA) containing the RNA-dependent RNA polymerase-encoding sequence of the virus was used as the reference standard. A series of five log_10_ dilutions corresponding to 1 x 10^1^ to 1 x 10^6^ copies per reaction of reference standard was run in parallel with the test samples.

### Virus Isolation

Specimens resuspended in virus transport medium (200 μL) were used for infecting fetal rhesus monkey kidney (FRhK-4) cell monolayers in culture tubes. Approximately 1 g of feces samples were resuspended in 10 mL virus transport medium and centrifuged, and the supernatant was spread onto cells. The respiratory samples were already diluted in virus transport medium and spread onto the cell monolayer. After incubation at 37°C for 1 h, the cells were fed with 1 mL of minimum essential medium with 1% fetal calf serum (GibcoBRL, Grand Island, NY) and incubated at 37°C. The cultures were examined for cytopathic effect (CPE) each day for 14 days. At the end of the incubation period or when CPE appeared, the cells were spotted on Teflon-coated slides, fixed with ice-cold acetone, and stained for SARS-CoV antigen by using a convalescent-phase human serum. The identification of the isolate was confirmed by RT-PCR.

### Serologic Testing

Coronavirus immunoglobulin G serologic testing was performed by indirect immunofluorescence. Batches of SARS-CoV–infected Vero cell smears were prepared and fixed in ice-cold acetone for 10 minutes. The cells were adjusted to be 60% to 70% SARS-CoV infected, as judged by immunofluorescent staining with a control positive human convalescent-phase serum. The fixed smears were stored at –70°C until use. Serum samples were screened at a dilution of 1:10 on infected and uninfected control cells. After 30 minutes of incubation, the cells were washed twice in phosphate-buffered saline (PBS) for 5 minutes each, and then goat anti-human fluorescein isothiocyanate conjugate (INOVA Diagnostics, Inc., San Diego, CA) was added, and the cells were incubated for 30 minutes at 37°C. The cells were washed again as described and examined with an immunofluorescent microscope. Serum samples positive at a screening dilution of 1:10 were titrated with serial twofold dilutions in parallel with the respective acute-phase serum specimen from the same patient. A positive control serum was tested with each batch of cells.

### Biosafety

Virus isolation or preparing cell smears for serologic testing was done in a biosafety level (BSL) 3 laboratory. Routine handling of clinical specimens for RNA extraction and serologic testing by immunofluorescence were done in a BSL-2 laboratory. Basic laboratory practice was reinforced by educating staff and closely supervising work practices. Serum specimens for antibody testing were heat inactivated at 56°C for 30 minutes before testing.

## Results

The sensitivity and specificity of the RT-PCR and the real time LightCycler assays have been reported (*9*,*11*,*12*). A total of 3,611 respiratory, fecal, and urine specimens and 1,699 serum samples were tested for SARS-CoV RNA and antibody, respectively, from 1,048 patients for whom an initial clinical suspicion of SARS was considered. The laboratory results were retrospectively correlated with the clinical diagnoses of these patients. Clinically, 590 of these patients were considered to have clinical SARS, 79 to have suspected SARS, and 379 not to have SARS. The third group included patients hospitalized with febrile respiratory illnesses, many with radiologic changes, in whom SARS had been initially considered in the differential diagnosis.

Overall, 948 (91%) of the patients had one or more specimens tested for SARS-CoV RNA by RT-PCR, and 454 (43%) had acute- and convalescent-phase serum samples available for serologic analysis, with a convalescent-phase serum taken at least 21 days after onset of illness. While specimens for RT-PCR were available from similar proportions (89%–91%) of patients in each clinical category, paired sera were more frequently available from patients clinically categorized as having SARS (417 [71%] from 590) than from patients in the not SARS category (25 [7%] from 379) (Table 1).

**Table 1:** SARS-CoV RNA detection by RT-PCR in clinical specimens^a^

Category	Patients tested	Patients positive (%)
Clinical		
Clinical SARS (n = 590)	537	322 (60.0)
Suspected SARS (n = 79)	70	1 (1.4)
Non-SARS febrile respiratory illnesses (n = 379)	341	2 (0.6)
Hospital controls		
Cohort 1: fecal samples from non-SARS patients with diarrhea	184	1 (0.5)
Community controls		
Cohort 2: throat swabs from patients with nonrespiratory illness visiting community physicians.	148	0 (0.0)

^a^SARS, severe acute respiratory syndrome; SARS-CoV, severe acute respiratory syndrome coronavirus; RT-PCR, reverse transcriptase–polymerase chain reaction.

Of the patients clinically diagnosed as having SARS, 322 (60%) of 537 patients had evidence of SARS-CoV RNA in clinical specimens. In contrast, 2 (0.6%) of 341 of those clinically diagnosed as the “not SARS” category had RT-PCR evidence of SARS-CoV infection (Table 1). To assess the extent of circulation of SARS-CoV in the general population, 184 fecal specimens (submitted for investigation of diarrheal illnesses from patients thought not to have SARS) and 148 nose and throat swabs (from patients visiting a general practice for nonrespiratory illnesses) were tested for viral RNA by RT-PCR. None of 148 control throat swab specimens and 1 of 184 control stool specimens had evidence of detectable SARS-CoV RNA.

Of 417 patients with clinical SARS for whom paired sera were available, 383 (92%) had a >4-fold rise in antibody titer to SARS-CoV. None of 45 controls had seroconversion to SARS-CoV. Two (8%) of 25 patients clinically diagnosed as the “not SARS” category seroconverted (Table 2), but a further 47 convalescent-phase sera from patients in this group failed to show any more seropositive patients (data not shown). Neither of these two patients had a history of contact with other patients with SARS. However, one had a left mid-zone consolidation confirmed by high-resolution computed tomography scan and had a discharge diagnosis of pneumonia of unknown cause. The other had a mild febrile illness of unknown cause without radiologic evidence of consolidation. None of 200 blood donor serum samples collected in Hong Kong during March 2003 and 2,200 additional serum samples collected in May 2003 had evidence of antibody to SARS.

**Table 2:** Serologic response to SARS coronavirus^a^

Clinical category	No. of patients	Paired sera available for study	No (%) of patients with 4-fold rise in antibody titer to SARS-CoV
Clinical SARS	590	417	384 (92.1)
Suspected SARS	79	11	1 (9.1)
Not SARS	379	25	2 (8.0)
Controls	45	45	0 (0.0)

^a^SARS, severe acute respiratory syndrome; SARS-CoV, severe acute respiratory syndrome coronavirus. ^b^An additional 47 convalescent-phase sera were subsequently tested without any further evidence of antibody to SARS-CoV.

The profile of SARS-CoV RNA detection in the 386 patients with serologically confirmed SARS-CoV infection was analyzed (Figure). Viral RNA was detectable in the respiratory tract of a proportion (11%–42%) of patients within the first 4 days of illness but was not detectable in stool or urine specimens until days 5 and 7 of the illness, respectively. The proportion of respiratory and stool specimens positive for viral RNA progressively increased and then peaked at approximately day 11 of the illness. While the nasopharyngeal aspirates and throat and nose swabs were the most productive specimens in the first 4 days of disease, stool samples were more useful after the 5th day of illness. Although the rate of detection in clinical specimens gradually decreased from day 16 onward, viral RNA could still be detected after 30 days of illness in samples from the nasopharynx, feces, and urine in a small proportion of patients (Figure). Smaller numbers of saliva, endotracheal aspirate, and sputum specimens were available for testing (Table 3).

**Figure:**
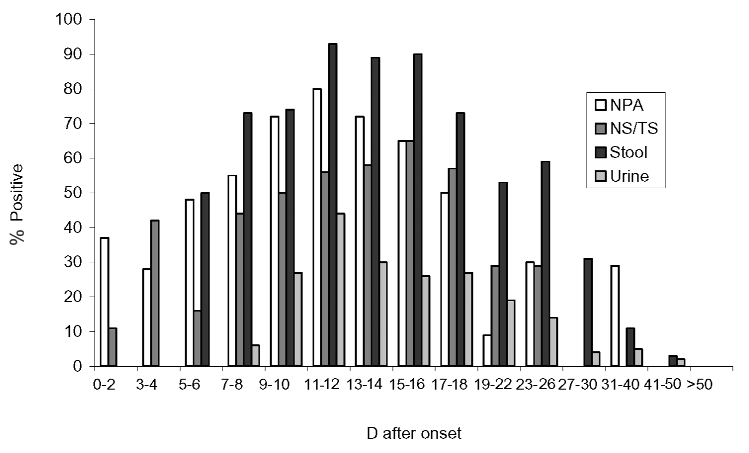
Reverse transcriptase–polymerase chain reaction percent positive in nasopharyngeal aspirates, nose and throat swabs, and stool and urine specimens at different days after onset of illness in patients with serologically confirmed severe acute respiratory syndrome. NPA, nasopharyngeal aspirate; NS/TS, nasal and throat swabs.

**Table 3:** SARS coronavirus RNA detection in saliva, endotracheal aspirates, and sputum at different times after onset of illness in patients with serologically confirmed SARS-CoV infection^a^

D after onset	Positive saliva samples/total (%)	Positive endotracheal aspirate/total (5)	Positive sputum/total
0–4	ND	ND	3/6
5–10	1/6 (17.0)	1/2	3/3
11–20	6/45 (13.3)	2/3	1/1
21–30	2/96 (2.1)	13/19 (68.4)	ND
31–40	3/58 (5.2)	1/1	ND
41–50	1/29 (3.4)	ND	ND
>50	0/40 (0.0)	0/1	0/1

^a^SARS, severe acute respiratory syndrome; SARS-CoV, severe acute respiratory syndrome coronavirus; ND, not done.

Since confirmation of a laboratory diagnosis of SARS within the first 5 days of illness is the greatest clinical need, we studied the diagnostic yield from different specimens in patients with serologically confirmed SARS-CoV infection during this period (Table 4). Sputum appeared to be a good clinical specimen in the early stage of the disease, although the number of specimens tested was small. Nasopharyngeal aspirates and throat and nose swabs appear to be of comparable sensitivity (30% and 28%, respectively), while stool specimens are less useful specimens in the first 5 days of illness (sensitivity 20%). Saliva and endotracheal aspirates are alternative specimens (Table 3), but we could not assess their usefulness because of the lack of specimens collected in the early stage of the illness. In patients whose first specimen tested negative, 25 had a second specimen (of any type) collected within the first 5 days of illness. Three of these 25 were positive; the additional diagnostic yield from a second specimen was approximately 12% (data not shown).

**Table 4:** RT-PCR for diagnosis of SARS-CoV in the first 5 days of illness in patients with serologically confirmed SARS-CoV infection^a^

Specimens evaluated	Positive/tested (%)
Nasopharyngeal aspirate	29/98 (29.6)
Swabs (throat, nose)	15/53 (28.3)
Sputum	5/9 (55.6)
Stool	5/25 (20.0)
Urine	0/15 (0.0)

^a^SARS, severe acute respiratory syndrome; SARS-CoV, severe acute respiratory syndrome coronavirus; RT-PCR, reverse transcriptase–polymerase chain reaction.

Virus was isolated retrospectively from stored clinical specimens that were RT-PCR positive for viral RNA (Table 5). Virus was more readily isolated from the respiratory tract than from stool specimens. Furthermore, virus isolation was most successful during the first 2 weeks of the illness and was generally negative after day 22 of illness, even though virus was detectable in these specimens by RT-PCR.

**Table 5:** Virus isolation from specimens positive for SARS-CoV by RT-PCR^a^

Wk	Sample type				
	Positive NPA/sputum/total (%)	Positive TS/total (%)	Positive stool/total (%)	Positive urine/total (%)	Total pos/total tested (%)
1	3/11 (27.3)	0/3 (0)	0/0 (0)	0/0 (0)	3/14 (21.4)
2	20/37 (54.1)	1/6 (16.7)	0/11 (0)	1/4 (25.0)	22/58 (37.9)
3	0/6 (0)	1/6 (16.7)	1/18 (5.6)	0/0 (0)	2/30 (6.7)
4	0/3 (0)	0/0 (0)	0/7 (0)	0/0 (0)	0/10 (0)
Total	23/57 (40.4)	2/15 (13.3)	1/26 (3.8)	1/4 (25.0)	27/112 (24.1)

^a^SARS, severe acute respiratory syndrome; SARS-CoV, severe acute respiratory syndrome coronavirus; RT-PCR, reverse transcriptase–polymerase chain reaction; NPA, nasopharyngeal aspirate; TS, throat swab.

## Discussion

In April 2003, the first-generation diagnostic tests for the SARS-CoV became available to clinicians caring for patients in whom SARS was considered in the differential diagnosis. Normally, new laboratory diagnostic tests are extensively evaluated and validated before they are introduced in routine clinical practice. However, in the case of SARS, a new and poorly understood disease, these first-generation test results were provided to clinicians on the understanding that the tests had not been validated and results had to be interpreted with caution.

Continued improvement of the sensitivity of RT-PCR methods (*12*) makes an analysis of the sensitivity of these first-generation diagnostic methods less relevant. However, these results provide useful information on the best specimens for detection of virus at different stages of illness, the tissue tropism of the virus, and the duration of virus excretion.

Culture of SARS-CoV for preparing the virus-infected cell smears and for virus isolation was carried out under BSL3 conditions, but routine clinical specimens were processed in the clinical virology laboratory under BSL2 conditions after enhanced and reinforced education on safety and good laboratory practice. Given that up to 250 specimens per day were being processed for RT-PCR detection and serologic testing during peak periods, the workload could not be managed in a BSL3 laboratory. None of the laboratory staff became ill with SARS symptoms, indicating that clinical specimens for serologic testing and RT-PCR can be processed safely in BSL2 level conditions.

The association of SARS-CoV with the clinical syndrome of SARS is illustrated by the detection rates of viral RNA in clinical specimens (60% in patients with SARS, 0.6% in the non-SARS group, and 0.3% of controls). Viral RNA detection by these first-generation RT-PCR tests is less sensitive than serologic testing for diagnosing SARS. Correspondingly, 92% of 417 patients with clinically diagnosed SARS and none of the paired sera from 45 unrelated controls seroconverted to SARS-CoV. However, 2 of 25 patients designated as “not SARS” category from whom paired sera were available also seroconverted. Paired sera were available from only a few (25 of 379) patients in the “not SARS” group. At a time of intense pressure on the clinical front-line staff, there was little incentive to obtain convalescent-phase sera from patients believed not to have SARS. These 25 patients may represent a biased sample of the larger group of non-SARS patients. This contention is supported by the fact that a further 47 convalescent-phase sera subsequently obtained from this group of “not SARS” patients failed to show any additional antibodies to SARS. Even patients in the “not SARS” category had a febrile, respiratory, often pneumonic, illness; one of the two patients in the “not SARS” category who had evidence of seroconversion had an undiagnosed pneumonic illness, while the other had an undiagnosed febrile illness without radiologic consolidation of the lung. Overall, a clinical diagnosis of SARS is closely correlated with detection of viral RNA by RT-PCR and seroconversion supporting the etiologic association of SARS-CoV and SARS.

None of 2,400 blood donor sera collected in Hong Kong during the height of the SARS outbreak has any evidence of antibody to the virus. This finding suggests that the spread of SARS-CoV infection in the general community was minimal, with most of the infection associated with clusters and hospital outbreaks (*13*).

The RT-PCR detection rates for SARS-CoV in respiratory, stool, and urine specimens in the 383 patients with seroconversion to SARS-CoV show that viral shedding progressively increased from onset of the illness until approximately day 11 after onset. Since the first-generation RT-PCR test has relatively low sensitivity, these results reflect the increasing viral load at different clinical sites during the illness. Whereas these data are cross-sectional, in a previous study viral load in nasopharyngeal aspirates was followed up longitudinally in nasopharyngeal specimens collected at days 5, 10, and 15 after illness onset; results of this study also indicated that viral load peaks at day 10 of illness (*4*). Such a profile of a progressive increase in viral load is unusual for respiratory viral infections. Most other infections (e.g., respiratory syncytial virus, influenza) have peak viral titers in the respiratory secretions at or soon after the onset of clinical illness, after which viral titers and laboratory diagnostic yield decrease progressively (*14*). This “crescendo” pattern in SARS-CoV detection rates and viral load in clinical specimens has a number of implications. The pattern explains the poor sensitivity of the first-generation diagnostic tests during the first 5 days of the illness and emphasizes the challenge in making laboratory diagnosis early in the disease. These results may also suggest a fundamental difference in the efficacy of the innate immune response in controlling SARS-CoV infection, in contrast, for example, with influenza infection. Innate immune mechanisms are the earliest host defenses that control viral replication and, in the case of many respiratory viruses, do so within the first few days of illness, even before the specific adaptive immune responses have been activated. This response does not appear to occur with SARS, and viral load in the respiratory tract (*4*) begins to fall only when the antibody response appears, i.e., at approximately day 10 after onset of illness (*4**,**5*). This finding may suggest that SARS-CoV is able to evade the host innate response and requires the adaptive immune response to bring the infection under control. Finally, the peak viral load in the 2nd week of illness would predict that virus is more likely to be transmitted later in the course of the illness. This result indeed accords with epidemiologic observations (*15*). With regard to observations of viral load, the frequent use of steroid therapy in hospitals (*16*) is a confounding factor that may contribute to the increase in virus load later in the illness.

The relative virus detection rates from different specimens during the illness suggests that respiratory specimens (nasopharyngeal aspirate, throat swab) are more useful in the first 4 days of the illness, while fecal samples are better later in the illness. Urine samples, on the other hand, are not useful at any stage of the illness. A productive cough is not common in the early stage of illness, but in patients who do produce sputum, this specimen provides a high diagnostic yield. Thus, nasopharyngeal aspirates, throat swabs, and sputum, if available, are the best specimens in the first 5 days of the illness.

Detecting virus in the fecal and urine samples, in addition to the respiratory tract, suggests that SARS is not restricted to the respiratory tract. The finding of diarrhea unrelated to antimicrobial drug use in a number of patients supports evidence that the disease is not a purely respiratory one (*4*). A number of animal coronaviruses (e.g., mouse hepatitis virus and feline coronavirus) have tropism for multiple organs (*17*). Viral shedding is detectable by RT-PCR in the respiratory, gastrointestinal, and urinary tracts for many weeks after onset of illness, reflecting continued virus replication at these sites. However, SARS-CoV cannot be readily cultured from any of these sites after week 3 of illness. The viral RNA detected by RT-PCR after week 3 of illness is unlikely to represent persistence of viral RNA in the absence of ongoing viral replication. The apparent dissociation between virus isolation and RT-PCR may reflect the mucosal antibody’s neutralizing the virus and rendering it less infectious. This observation also accords with the apparent absence of transmission of infection after week 2 of illness. The fact that virus isolation was done retrospectively may have affected the overall isolation rate. However, SARS-CoV appears relatively stable to freezing and thawing and is stable for many weeks in clinical specimens at 4°C or frozen at –70°C (K.H. Chan and J.S.M. Peiris, unpub. data). In any event, such a bias would be expected to be uniform both early and late in the disease.

In summary, SARS is closely associated epidemiologically with the novel SARS-CoV. The unusual profile of viral shedding from the respiratory tract may explain some of the observed transmission pattern of this disease, including the predilection for affecting healthcare workers.
